# Search for ferromagnetic order in overdoped copper-oxide superconductors

**DOI:** 10.1038/srep45896

**Published:** 2017-04-05

**Authors:** J. Wu, V. Lauter, H. Ambaye, X. He, I. Božović

**Affiliations:** 1 Brookhaven National Laboratory, Upton, NY 11973, USA; 2 Quantum Condensed Matter Division, Neutron Sciences Directorate, Oak Ridge National Laboratory, Oak Ridge, TN 37831, USA; 3 Instrument and Source Division, Neutron Sciences Directorate, Oak Ridge National Laboratory, Oak Ridge, TN 37831, USA; 4 Applied Physics Department, Yale University, New Haven, CT 06520, USA

## Abstract

In copper-oxides that show high-temperature superconductivity (HTS), the critical temperature (*T*_*c*_) has a dome-shaped doping dependence. The cause of demise of both *T*_*c*_ and superfluid density *n*_*s*_ on the overdoped side is a major puzzle. A recent study of transport and diamagnetism in a large number of overdoped La_2−x_Sr_x_CuO_4_ (LSCO) films shows that this cannot be accounted for by disorder within the conventional Bardeen-Cooper-Schrieffer theory. This brings to focus an alternative explanation — competition of HTS with ferromagnetic order, fluctuating in superconducting samples and static beyond the superconductor-to-metal transition. Here, we examine this proposal by growing single-crystal LSCO thin films with doping on both sides of the transition by molecular beam epitaxy, and using polarized neutron reflectometry to measure their magnetic moments. In a heavily overdoped, metallic but non-superconducting LSCO (*x* = 0.35) film, the spin asymmetry of reflectivity shows a very small static magnetic moment (~2 emu/cm^3^). Less-doped, superconducting LSCO films show no magnetic moment in neutron reflectivity, both above and below *T*_*c*_. Therefore, the collapse of HTS with overdoping is not caused by competing ferromagnetic order.

The superconducting temperature *T*_*c*_ in cuprates shows an unusual dome-shaped dependence on the doping level, which presumably originates from an unconventional pairing mechanism. On the low-doping side of the dome in the *x-T* (doping-temperature) phase diagram, the competition of HTS with antiferromagnetism, charge density waves, and the pseudogap phase has been investigated extensively although some details are still debated^1^,^2^. The overdoped side has been studied less and the situation is even more puzzling. It has been speculated that the demise of *T*_*c*_ and the superfluid density *n*_*s*_ with overdoping originates from pair breaking caused by impurities, disorder, and phase separation, which could be accounted for within the conventional dirty-BCS (Bardeen-Cooper-Schrieffer) picture^3^. However, this hypothesis has been thoroughly scrutinized and refuted in the recently released detailed study of penetration depth and magnetoresistance in a huge number of overdoped LSCO films^4^ grown by atomic-layer-by layer molecular beam epitaxy (ALL-MBE). In these high-quality single-crystal films, the superfluid seems to be homogeneous and uniform at every doping.

This brings to the forefront an alternative explanation that on the high-doping side HTS competes with ferromagnetic order^5^. In fact, ferromagnetism does occur in closely related oxides such as La_1−x_Sr_x_CoO_3_ (ref. 6). Electronic band calculations show a tendency towards ferromagnetic ordering^7^ at high charge doping in La_2−x_Ba_x_CuO_4_. The dramatic evolution of Fermi surface with overdoping in LSCO may provide a chance for magnetic moments to be interacting and correlated in a preferred direction, in some composition window. Several experimental observations provide some support to this scenario^8^,^9^,^10^,^11^,^12^,^13^,^14^,^15^,^16^. The temperature dependence of magnetic susceptibility (*χ*) in overdoped LSCO crystals indicated the absence of local magnetic moments below the critical doping *x*_*c*_ ≈ 0.18, and dramatic increase in their density with further overdoping^8^,^9^,^10^,^11^,^12^. Muon spin rotation (μSR) experiments^15^ detected the onset of static magnetic moments at low temperature in heavily overdoped (*x* = 0.33) LSCO, metallic but non-superconducting (in what follows, we refer to such samples simply as ‘metallic’). However, no ferromagnetism has been detected so far in the vicinity of the quantum (*T* = 0) superconductor-to-metal transition (SMT) that occurs at the overdoped dome edge (*x*_*c2*_ ≈ 0.26).

## Experimental

With this motivation, we used polarized neutron reflectometry (PNR) to search for magnetic order in single-crystal films of overdoped LSCO, covering both the superconducting and metallic states. PNR yields model-free information on both the absolute value of magnetic moments down to ~1 emu/cm^3^ (ref. 17) and on the magnetic order^18^,^19^,^20^ with the depth-resolution of 0.5 nm. To achieve the best sensitivity PNR experiments require thin films of excellent crystallinity, with atomically flat surfaces and interfaces. More important, one should minimize the density of structural defects and oxygen vacancies, which are known to build up with increased Sr doping on the overdoped side^21^. Such extrinsic defects may polarize nearby electrons and introduce additional magnetic signals^22^ that are difficult to distinguish from intrinsic phenomena we are interested in. For these reasons, the film quality is crucial for this type of experiments.

For film synthesis, we have used the advanced ALL-MBE system at Brookhaven National Laboratory (BNL). It produces HTS single-crystal thin films with state-of-art structural quality, extremely sharp superconducting transitions, and very low density of defects inferred from the small residual resistivity^23^. Our LSCO thin films are grown on single-crystal LaSrAlO_4_ (LSAO) substrates. Because of the lattice mismatch with LSAO, our LSCO films are under compressive epitaxial strain, and for this reason SMT occurs at the critical doping *x*_*c2*_ ≈ 0.30, slightly larger than in bulk LSCO^24^. Hence, we chose the Sr doping level *x* = 0.27, 0.29 and 0.35 to cover both the superconducting and metallic states in the vicinity of SMT. Note that the Curie-like magnetic susceptibility peaks at *x* = 0.30 in bulk LSCO^12^, which corresponds to *x* = 0.35 in our thin films due to the epitaxial strain; therefore the ferromagnetic order should be at the maximum at *x* = 0.35, if it exists. After the deposition we anneal the films *in situ* in atmosphere of pure ozone, in order to minimize the density of oxygen vacancies. Compared to bulk crystals, the oxidation in thin films can be fully controlled and they can be fully oxidized more easily. For example, in bulk La_2_CuO_4+δ_ crystals the largest value of δ corresponded to a mixture of staging numbers 2, 3, and 4 (meaning that every second, third, or fourth layer, respectively, contained extra interstitial oxygen)^25^,^26^. And even this was only achieved by electro-chemical oxidation; annealing in oxygen atmosphere, even at high pressure, is generally much less effective. In contrast, in ultrathin films, by annealing in ozone atmosphere, we easily achieve *n* = 1, with a much higher interstitial oxygen content. This difference is attributed largely to the epitaxial strain, although the high oxidation power of ozone also plays a role^24^

Superconducting transitions in these films were determined at Brookhaven National Laboratory by the two-coil mutual inductance measurements^4^,^27^,^28^. PNR experiments were performed at the Magnetism Reflectometer at Spallation Neutron Source (SNS) in Oak Ridge National Laboratory (ORNL)^29^. The high polarization (98.5%) of the neutron beam and a very low instrumental background of this reflectometer are essential for reaching the sensitivity necessary to detect small magnetic moments. During the cool down process of the sample from 300 K to the measurement temperature, a 1.15 T magnetic field is applied in-plane of the film and parallel to the CuO_2_ planes. This field-cooling process helps to overcome magnetic pinning from impurities or defects, and to align the magnetic moments in the desired direction. This 1.15 T field is applied during the measurements as well to keep the magnetic moments aligned, and generate the maximum contrast of spin asymmetry in neutron reflectivity.

More details on film synthesis, characterization and neutron experiments are described in the Methods section.

## Results

**Figure 1:**
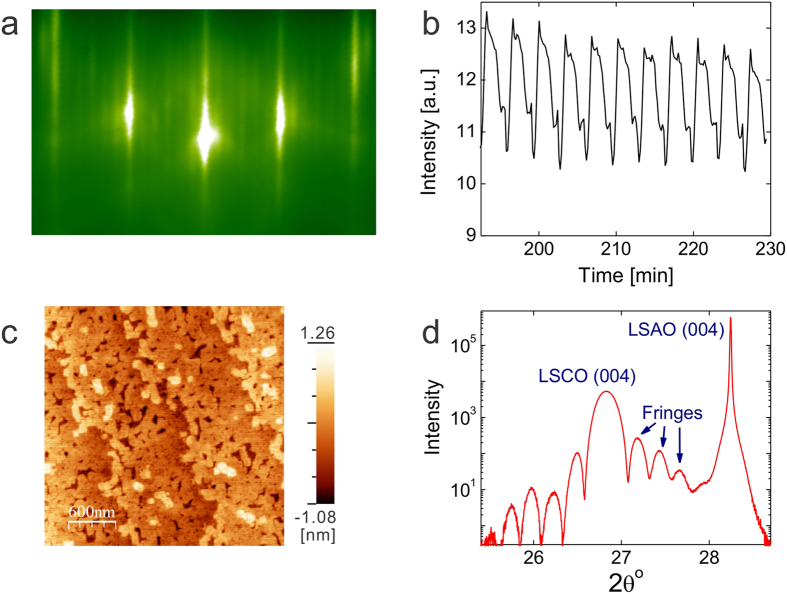
Evidence for a high quality of LSCO films synthesized by ALL-MBE. (**a**) A reflection high-energy electron diffraction (RHEED) pattern taken from a 30 UC thick overdoped LSCO film shows strong specular reflection, surface reconstruction that occurs after CuO_2_ planes, and clear Kikuchi lines, indicating a very high crystalline coherence. (**b**) The last ten periods of the intensity oscillations of the RHEED specular spot. (One period corresponds to deposition of one complete LSCO monolayer). The amplitude of oscillations does not decay till the end of deposition, indicating essentially perfect 2D growth. (**c**) The atomic force microscopy (AFM) image taken from the same film after growth. Clear atomic steps are imprinted from the LSAO substrate and still visible on the top layer of LSCO. The root-mean-squared (RMS) surface roughness is 0.29 nm, much less than 1 UC height. (**d**) The X-ray diffraction spectrum taken from the same sample illustrates high crystalline quality and sharp LSCO/LSAO interface evidenced by the well-defined Laüe fringes.

In Fig. 1a, we show representative reflection high-energy electron diffraction (RHEED), image taken during an LSCO film growth. The sharp diffraction pattern, together with clear inelastic scattering (Kikuchi) lines, indicates high crystallinity and an atomically smooth film surface. RHEED is used to monitor every step of the film growth in real time, and in particular to study the oscillations in the intensity of the specular spot (Fig. 1b) that originate from the contrast between completely-filled (atomically smooth, maximal specular reflection) layers and half-filled (‘rough’, maximal diffuse scattering) layers. RHEED oscillations persist throughout the entire film growth, and even more remarkably, the amplitude of oscillations does not decay till the end, indicating essentially ideal perfect layer-by-layer growth. The number of oscillation periods provides accurately the information on the film thickness expressed as the number of unit cells. Together with the value of the *c*-axis lattice constant of 1.32 nm determined by X-ray diffraction^30^, this eliminates uncertainties related with the film thickness in the analysis of PNR experiments.

**Figure 2:**
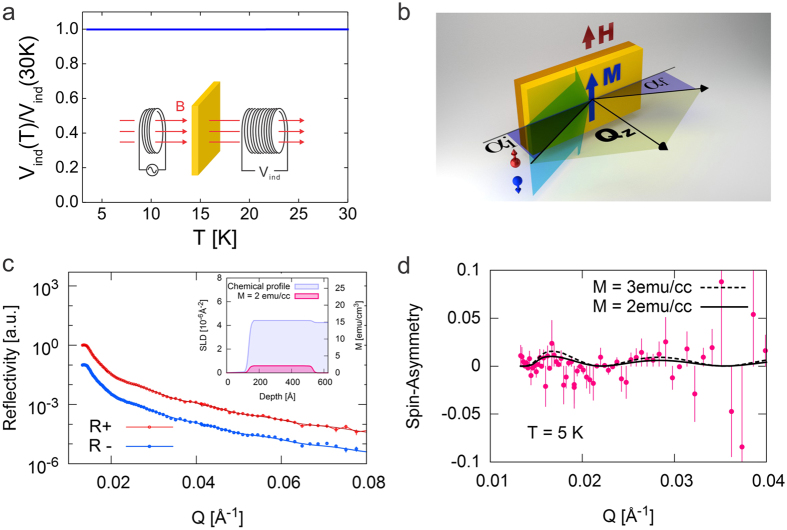
A heavily overdoped LSCO (x = 0.35) is metallic but not superconducting and possess a magnetization of ~2 emu/cm^3^. (**a**) The mutual inductance between two coils shows no diamagnetic shielding of the ac magnetic field by the inserted LSCO film, confirming that the film is not superconducting. The inset: a sketch of the two-coil mutual inductance measurement setup. (**b**) PNR scattering geometry. The collimated neutron beam with the neutron spin parallel (red sphere with arrow) and anti-parallel (blue sphere with arrow) to the applied external field *H* impinges onto the sample surface at a grazing incident angle *α*_*i*_ and specularly reflects at an angle *α*_*r*_, so that *θ*_*i*_ = *θ*_*f*_. The depth sensitivity of PNR offers the advantage of isolating and quantifying the magnetization of the LSCO film and its depth profile, unlike in conventional magnetometry methods that cannot disentangle possible substrate contributions. (**c**) The neutron reflectivity are separated for clarity by ×10 and plotted as a function of the momentum transfer for spin up, *R*^+^(*Q*), (red dots) and spin down, *R*^−^(*Q*), (blue dots), respectively. The red and blue solid lines are the *R*^+^(*Q*) and the *R*^−^(*Q*) curves, respectively, calculated based on chemical and magnetic profiles as shown in the inset; they match well the experiment data (red and blue dots). The inset: the depth profile of chemical (blue area) and magnetic (pink area) distributions that produce the best fits for *R*^+^(*Q*) and *R*^−^(*Q*). (**d**) The *Q*-dependence of the spin asymmetry can be accounted for by assuming a static magnetization of ~2 emu/cm^3^ in this LSCO film. Attempts to assume a larger magnetization, e.g., 3 emu/cm^3^ (the dashed line) make the calculated curve deviate notably from the experimental data.

Figure 1c depicts an atomic force microscopy (AFM) image taken from the surface of a 30 unit cell (UC) thick LSCO film. The local rms surface roughness is only 0.29 nm, much less than the unit-cell height. Apart from the steps and terraces that originate from the unavoidable small miscut of the substrate from the ideal crystallographic plane, the surface is atomically flat. Such structurally perfect single-crystal films provide an ideal platform for PNR experiments and analysis. Neutrons interact with the potential U^±^(z) = (2πћ^2^/m) (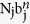 ± 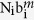) of the sample, where *m* denotes the neutron mass, while 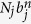 and 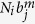 are the nuclear and magnetic scattering length densities (NSLD and MSLD, respectively). *N*_*j*_ is the atomic number density, while 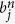 and 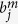 are the nuclear and magnetic scattering lengths. PNR is depth-sensitive technique. The depth profiles of the NSLD and MSLD correspond to depth profiles of chemical and *in-plane* magnetization vector distributions, respectively. It is obtained from a simultaneous fit to the neutron reflectivity data measured with neutron spin polarization parallel *R*^+^(*Q*) and antiparallel *R*^−^(*Q*) to the direction of the external magnetic field. Here *Q* is the wave vector transfer between the incoming and reflected neutrons with wave vectors *k*_*i*_ and *k*_*f*_, respectively; for an elastic scattering process, it is determined as *Q* = 2*k*sin*α*_*i*_ where *k* = 2π/*λ* with *λ* denoting the neutron wavelength and *α*_*i*_ the incident angle. Figure 2b shows a schematic of the experimental geometry. From the simultaneous fit of reflectivities *R*^+^ and *R*^−^, measured with the spin-up and spin-down neutron beam, the depth profile of the magnetization and the structure of the films are obtained. Modeling of the reflectivity data is performed using the well-established supermatrix formalism described in details earlier^31^,^32^. The parameters are refined by means of fitting by Levenberg-Marquardt algorithm^33^.

We first checked the proposal that static ferromagnetic order should occur in the vicinity of the SMT critical point^5^. In Fig. 2, we show the data taken from a metallic LSCO film with *x* = 0.35. The mutual inductance data (Fig. 2a) show no signature of the Meissner effect, confirming that the film is not superconducting, as expected. To explore the possible ferromagnetic order and to directly probe the magnetization depth profile we performed PNR experiments. Figure 2c shows experimental reflectivity profiles *R*^+^(*Q*) and *R*^−^(*Q*) for LSCO film with *x* = 0.35. Since the thermal energy tends to randomly flip the magnetic moments and weaken magnetic order, in order to maximize the magnetic contrast (if magnetic order indeed occurs), the sample temperature was kept at *T* = 5 K, the lowest that could be reached in the experimental setup. In Fig. 2c, on top of the fast decay of reflected intensity one can notice low-amplitude periodic oscillations in reflectivity as *Q* increases, for both spin polarizations. The oscillations are a result of the interference of the neutron waves reflected from the LSCO/LSAO and LSCO/vacuum interfaces. The period and amplitude of these oscillations are determined by the thickness of the film and the contrast between the film and substrate scattering length density (SLD).The structural and magnetic depth profiles of the film and the interfaces obtained from the simultaneous fit to the data^17^ are sketched in the insert of Fig. 2c. The substrate part of the model was first refined with a reference sample measurement and later kept constant. The LSCO films data could be refined with a single homogeneous layer and the film NSLD profile that confirms the excellent quality of the films. The film thickness is determined to be 39.6 nm, which is equivalent to 30 UC, in excellent agreement with the count from RHEED oscillations recorded during the film growth. The *R(Q*) curves also provide information on the morphology of the LSCO film surface, and indicate the surface roughness of 1.1 nm. This value is bigger than that retrieved from AFM and the difference comes from the uneven height across the measured area, resulting from the titling of the surface from (001) surfaces due to inevitable small miscut angle (<1°) in the substrate. PNR includes this as a contribution to roughness while AFM does not. No inter-diffusion is detected at the LSCO/LSAO interface to within less than 1 UC scale, in agreement with the evidence of a sharp interface from the Kiessig fringes observed in X-ray reflectivity and from the Laüe oscillations in X-ray diffraction measurements performed on the same sample (Fig. 1d).

The difference between *R*^+^(*Q*) and *R*^−^(*Q*) is determined solely by the magnetization in the film. Thus the spin asymmetry function, defined as *SA* = [*R*^+^(*Q*) − *R*^−^(*Q*)]/[*R*^+^(*Q*) + *R*^−^(*Q*)], allows to separate nuclear and magnetic scattering and serves as an efficient tool to detect magnetic moments^17^. The *SA* is equal to zero when no net magnetic moment is detected in the film. In Fig. 2d, the spin asymmetry in the metallic *x* = 0.35 LSCO film shows an oscillatory *Q* dependence, which is precisely reproduced in the fitted *SA* shown with the solid line in Fig. 2d. It corresponds to the magnetic depth profile depicted in the insert of Fig. 2c, and the best fit obtained with (volumetric) magnetization of 2 emu/cm^3^. If we assume a larger magnetization, e.g., 3 emu/cm^3^, the calculated asymmetry deviates notably from the experimental data, indicating that our experimental resolution is better than 1 emu/cm^3^, estimated as the minimum magnetization detectable within the statistical error bars of the experimental data. This corresponds to a very small magnetic moment of ~0.01 *μ*_*B*_ (the Bohr magneton) per Cu atom, as we expound in Discussion section.

**Figure 3:**
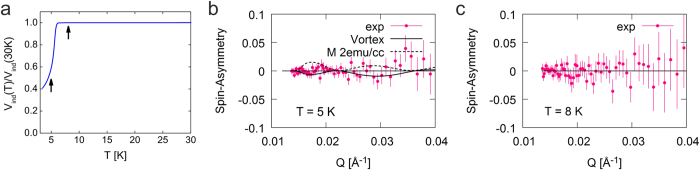
Overdoped LSCO (x = 0.29) film is superconducting (T_c_ = 6.3 K) and has no magnetization either above or below T_c_. (**a**) Due to Meissner effect, the superconducting film screens the magnetic field between the two coils and reduces their mutual inductance. *T*_*c*_ = 6.3 K is determined as the temperature at which the induced voltage *V*_*ind*_ starts to drop. The black arrows indicate the temperatures at which the PNR experiments were performed, covering both the superconducting and the normal state. (**b**) The spin asymmetry measured at 5 K is best fit by assuming zero magnetization. For comparison, the black dashed line is the spin asymmetry calculated assuming 2 emu/cm^3^ magnetization, which deviates significantly from the experimental data. Another possible scenario that corresponds to the mixed state in the sample is shown with black solid line. (**c**) The spin asymmetry at 8 K (i.e., in the normal state) indicates zero magnetization as well.

To investigate the magnetic moments in superconducting films, we studied an LSCO film with doping *x* = 0.29 and *T*_*c*_ = 6.3 K (Fig. 3a). To check the possibility whether magnetic signal coexist with superconductivity, we first performed PNR experiments at *T* = 5 K, probing the superconducting state (Fig. 3b). Then we repeated the measurements at *T* = 8 K, probing the normal state of the same film (Fig. 3c). In the normal state. we observe no magnetic contrast in the neutron reflectivity; the measured spin asymmetry data points are randomly distributed around zero within the experimental uncertainty. The best fit to these curves is obtained by assuming zero magnetization; given our instrument sensitivity, we conclude that the upper limit is 1 emu/cm^3^. To substantiate that, in Fig. 3b we also show the spin asymmetry (dashed lines) simulated while assuming a bigger magnetization of 2 emu/cm^3^; it evidently deviates from the experimental data points. Here we would like to point out that in the superconducting Meissner state below the lower critical field *H*_*c1*_ and in the mixed state above *H*_*c1*_ the magnetic field penetration can be measured using PNR as was successfully demonstrated in earlier experiments^34^,^35^,^36^. Although this topic goes beyond the scope of our paper and needs dedicated experiments, we attempted here to illustrate the mixed state with a model calculation^36^ shown in Fig. 3b. The external magnetic field 1.15 T applied during experiment is at least two orders of magnitude higher than *μ*_*0*_*H*_*c1*_ at this doping level, inducing superconducting vortex in the film. Under the assumption that 1.15 T field is still below the upper critical field *μ*_*0*_*H*_*c2*_, the mixed state was modeled with one, two, three and four vortex rows (see for details ref. 36). The best match to the data was obtained with one vortex row in the middle of the film (black solid line in Fig. 3b). The agreement of the fitting and the data at the low Q range is indicative of the mixed-state scenario that awaits a more conclusive experimental proof.

**Figure 4:**
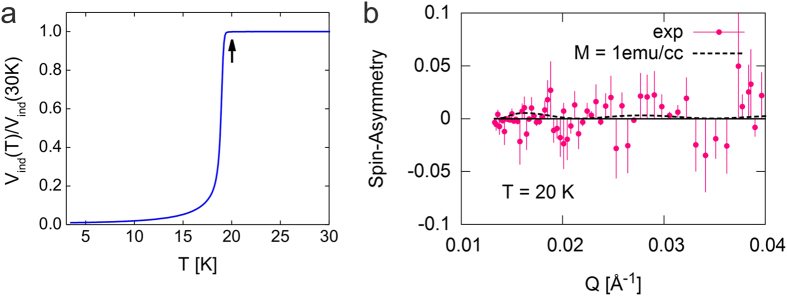
Overdoped LSCO (x = 0.27) film (T_C_ = 19.3 K) is superconducting and has no static magnetization. (**a**) The mutual inductance data showing *T*_*c*_ = 19.3 K. (**b**) The best fit to the measured spin asymmetry is obtained assuming zero magnetization. The curve assuming 1 emu/cm^3^ magnetization (dashed line) deviates from the data modestly that marks the sensitivity of our measurements.

Moving further into the superconducting state, we performed measurements on another LSCO film with still lower doping level (*x* = 0.27) and a higher *T*_*c*_ = 19.3 K (Fig. 4a). The best fit to the spin asymmetry at *T* = 20 K gives a zero magnetic moment (Fig. 4b); thus no magnetic order was detected in this superconducting film either.

Based on extensive transport measurements on a large set of LSCO films, SMT occurs at the critical doping *x*_*c2*_ ≈ 0.30. The ground state of LSCO overdoped beyond *x*_*c*_ is metallic, with a magnetization of ~2 emu/cm^3^. If the doping level is reduced to less than *x*_*c2*_, LSCO becomes superconducting and shows no magnetization either below and above *T*_*c*_. (Note that we are measuring at the magnetic field *B* = 1.15 T, orders of magnitude higher than *μ*_*0*_*H*_*c1*_). There we can put an upper limit of magnetization at 1 emu/cm^3^, which is the sensitivity of the PNR experiment.

## Discussion

To interpret our observations, we should identify and distinguish different sources of magnetic contrast in the PNR experiments. The PNR signal includes contribution from all types of magnetic moments that are completely or partially aligned in the direction of the external field, and hence, paramagnetic spins would contribute to the measured spin asymmetry. The size of this contribution can be estimated from the measured magnetic susceptibility. In overdoped LSCO (*x* = 0.33), the paramagnetic susceptibility^12^,^15^ (including Pauli, Curie and other terms) is *χ* = 4 × 10^−6^ emu/cm^3^ at T = 5 K. The corresponding magnetization in the applied 1.15 T magnetic field is ~0.05 emu/cm^3^, which is ~40 times smaller than the magnetization we measured in the *x* = 0.35 LSCO film (~2 emu/cm^3^). Therefore we conclude that the paramagnetic effect is not very important for our observations; the magnetic contrast for the most part originates from static magnetic moments.

The next question is about the spatial distribution of these moments. One proposed scenario assumes that every Cu^2+^ ion provides the same contribution to the net magnetization. In this case the measured magnetization of *M* = 2 emu/cm^3^ would imply that every Cu^2+^ ion carries the magnetic moment of 0.02 *μ*_*B*_ (Bohr magneton). The exchange interaction among such small moments would be too weak for long-range magnetic order to form. Then, to account for the fact that these magnetic moments keep a fixed orientation and are stable against thermal fluctuations, one wound need to postulate that there is a pinning center next to every Cu^2+^ ion, but this is highly unlikely.

A more likely alternative is that magnetic moments are larger but sparse and randomly distributed across the film. If we assume that the magnetic moment is 1 μ_B_, then in our *x* = 0.35 LSCO film the density of such spins would be ~2% (0.02 per one Cu atom). Such low spin concentration would point to their extrinsic origin, e.g., related to oxygen vacancies. (Magnetic atom impurities can be ruled out since their presence is negligible in our MBE-grown films; the purity of source metals we use is better than 99.9% for La and Sr and better than 99.999% for Cu.) The density of oxygen vacancies is known to increase with Sr doping in the heavily overdoped region^21^. Our state-of-the-art ALL-MBE synthesis of ultrathin LSCO films combined with post-growth ozone-annealing process may currently be the best approach to minimize the density of oxygen vacancies. Nevertheless, since a highly accurate method to measure the oxygen vacancy density in such thin films is not available, we cannot rule out the possibility that even after ozone annealing in the *x* = 0.35 LSCO film we still have ~2% of residual oxygen vacancies. While they may not carry magnetic moment by themselves, they could pin nearby paramagnetic moments of Cu^2+^ ions, making them static. This scenario is similar to doping Zn into overdoped LSCO where every Zn atom induces an additional 1.2 μ_B_ moment^11^. The difference between these two cases is that oxygen vacancies may pin these moments to the magnetic field direction during the field-cooling process, making the moments stable against thermal fluctuations.

Our proposed interpretation of the PNR results is consistent with the findings in other reported studies of the magnetic state in overdoped HTS materials. Measurements of the magnetic susceptibility *χ* in overdoped LSCO and Tl-2201 [refs 8, 9, 10, 11, 12] revealed the appearance of an additional term proportional to 1/*T*. This Curie-like paramagnetic susceptibility originates from localized magnetic moments that are fluctuating at finite temperature and can be polarized by a magnetic field. The coefficient of the Curie term increases rapidly with doping for *x* > *x*_*c*_ (for LSCO, *x*_*c*_ = 0.18) and eventually starts to decrease gradually above *x* = 0.30. The estimated density of these local moments is on the same order of magnitude as the density of static moments detected in our PNR experiment. Next, μSR measurements on LSCO single crystals revealed a low concentration of static magnetic moments at doping *x* = 0.33 [ref. 15], in agreement with our findings. The fact that consistent results are obtained by independent methods, on different types of samples (bulk crystals versus thin films), and in different materials suggests that this may be generic behavior of heavily overdoped, non-superconducting copper-oxide compounds.

When Sr doping is reduced and LSCO films become superconducting, the magnetic state changes dramatically. Below superconducting *T*_*c*_, there is no sign that superconducting state coexist with magnetic moments (Fig. 3b). In the superconducting state, Cooper pairs with opposite spins propagate through the lattice and on their way tend to randomize local magnetic moments of Cu^2+^ ions, if any are present, and results in zero net magnetization. This may be the explanation of the fact that magnetic susceptibility showed sizable amount of paramagnetic moments in the 0.18 < *x* < 0.26 doping range, while our PNR experiment did not detect any static magnetic order for comparable doping. In Figs 3c and 4b we show that static magnetic moments are absent even above *T*_*c*_ where superconductivity loses ground to the metallic normal state. This may be explained in the same way, since massive superconducting fluctuations persist well above *T*_*c*_ [ref. 37] and they could very effectively suppress the static magnetic order between local moments. An alternative possibility is that the key factor is oxygen vacancies, and that their density in our ozone-annealed ultrathin films is significantly lower than in bulk crystals.

In summary, we have searched for and have not observed a static magnetic order in the vicinity of SMT strong enough to account for the demise of *T*_*c*_ and superfluid density on the overdoped side of the LSCO phase diagram. In metallic but non-superconducting LSCO films we observed a very small static moment; this is significant insofar that it nicely demonstrates the sensitivity of the technique — ferromagnetism is detected when it is present, even if it is weak or existent in only a small fraction of the sample. In less-doped, superconducting LSCO films no static moments are seen, either above or below *T*_*c*_. Hence, we found out that the demise of HTS with overdoping cannot be attributed to competition with ferromagnetism.

## Methods

### Film synthesis

LSCO thin films were deposited onto LSAO substrates by ALL-MBE. The crystal structure of the films was monitored in real time by RHEED. The intensity of the specular spot oscillates, reaching the maximum upon completion of LSCO monolayers, thus providing accurate information on the film thickness. The film morphology is characterized by AFM, and the typical roughness is less than 0.3 nm, i.e., the films are atomically smooth. The same conclusion is inferred from XRD and X-ray reflectance measurements, and is consistent with structural information retrieved from PNR measurements.

### Mutual inductance

The mutual inductance between two coils was measured by exciting the driving coil with ac (40 kHz) current and measuring the ac voltage on the pick-up coil by means of a lock-in amplifier (see the insert of Fig. 2a). The amplitude of the ac excitation current was 40 μA, generating the magnetic field of amplitude less than 0.01 Gauss, much smaller than the lower critical magnetic field (*H*_*c1*_) of the LSCO film. The gap between the two coils is 2 mm and LSCO films are inserted into the gap. The whole setup is integrated into a sample stage mounted onto the cold finger of a Helium close-cycle cryocooler, and the temperature can be varied from 3 K to 300 K^38^. When the film is cooled down below *T*_*c*_, it screens the magnetic field and the mutual inductance drops rapidly, providing a sensitive probe of *T*_*c*_ in our films.

### Polarized Neutron Reflectometry

The neutron reflectivity experiments were carried out at the Magnetism Reflectometer at the Spallation Neutron Source (SNS) in Oak Ridge National Laboratory (ORNL)^29^. Neutrons with a high polarization of 99% to 98.5% with wavelengths within a band *Δλ* of 2 to 8 Å were used. Measurements were performed in a closed cycle refrigerator (Advanced Research System CCR) with an applied external magnetic field by using a Bruker electromagnet with a maximum magnetic field of 1.15 Tesla. Using the time-of-flight method, a highly-collimated polychromatic beam of polarized neutrons with the wavelength band *Δλ* impinges under grazing incidence on the film, where it interacts with atomic nuclei and the spins of unpaired electrons (see illustration in Fig. 2b). Being electrically neutral, neutrons are highly penetrating the entire multilayer structures and probe magnetic and structural composition of the film through the buried interfaces down to the substrate. The reflected intensity is measured as a function of the momentum transfer, *Q* = 4πsin*θ*/*λ*, for two neutron polarizations *R*^+^ and *R*^−^, with the neutron spin parallel (+) or antiparallel (−) to the direction of the external field, *H*_*ext*_. In order to magnify the magnetic scattering and to separate nuclear and magnetic components, the spin-asymmetry *SA* = (*R*^+^ − *R*^−^)/(*R*^+^ + *R*^−^) is used as depicted in Fig. 2d. A value of *SA* = 0 signifies zero net magnetic moment in the system.

## Additional Information

**How to cite this article**: Wu, J. *et al*. Search for ferromagnetic order in overdoped copper-oxide superconductors. *Sci. Rep.* **7**, 45896; doi: 10.1038/srep45896 (2017).

**Publisher's note:** Springer Nature remains neutral with regard to jurisdictional claims in published maps and institutional affiliations.
